# Variation in United States COVID-19 newborn care practices: results of an online physician survey

**DOI:** 10.1186/s12887-022-03129-0

**Published:** 2022-01-21

**Authors:** Margaret G. Parker, Arun Gupta, Helen Healy, Aviel Peaceman, Stephen M. Kerr, Timothy C. Heeren, Mark L. Hudak, Munish Gupta

**Affiliations:** 1 Department of Pediatrics, Boston Medical Center, Boston University School of Medicine, 801 Albany St, Room 2010, Boston, MA 02446 USA; 2 Department of Pediatrics, Stanford University School of Medicine, Palo Alto, USA; 3 Division of Neonatology, Boston Children’s Hospital, Harvard Medical School, Boston, USA; 4 Slone Epidemiology Center, Boston University School of Medicine, Boston, USA; 5 Department of Biostatistics, Boston University School of Public Health, Boston, USA; 6 Department of Pediatrics, University of Florida Health, University of Florida College of Medicine, Jacksonville, USA; 7 Department of Neonatology, Beth Israel Deaconess Medical Center, Harvard Medical School, Boston, USA

**Keywords:** COVID-19, Newborn care, Hospital care, Breastfeeding

## Abstract

**Background:**

Newborn care practices that best promote the health and well-being of mother-infant dyads after birth while minimizing transmission of COVID-19 were uncertain at the onset of the COVID-19 pandemic.

**Objective:**

Examine variation in COVID-19 newborn care practices among U.S. birth hospitals and by hospital characteristics (U.S. census region, highest level of neonatal level of care, and Baby-Friendly hospital status).

**Study Design:**

We surveyed physicians via American Academy of Pediatrics email listservs and social media between 5/26/2020-6/8/2020. Physicians identified the birth hospital in which they provided newborn care and their hospital’s approach to obstetrical and newborn care related to COVID-19. Chi-square tests were used to examine variation in hospital practices by U.S. census region, highest level of neonatal care, and Baby-Friendly hospital status.

**Results:**

Four hundred thirty three physicians responded from 318 hospitals across 46 states. Variation in care of SARS-CoV-2 positive mother-infant dyads was greatest for approaches to location of newborn care (31% separation, 17% rooming-in, and 51% based on shared-decision making), early skin-to-skin care (48% prohibited/discouraged, 11% encouraged, and 40% based on shared-decision making) and direct breastfeeding (37% prohibited/discouraged, 15% encouraged, and 48% based on shared-decision making). Among presumed uninfected dyads, 59% of hospitals discharged at least some mother-infant dyads early. We found variation in practices by U.S. census region.

**Conclusion:**

Approaches to newborn care and breastfeeding support for mother-infant dyads with positive SARS-CoV-2 testing differed across U.S. birth hospitals during the COVID-19 pandemic. Early discharge of presumed uninfected mother-infant dyads was common.

**Supplementary Information:**

The online version contains supplementary material available at 10.1186/s12887-022-03129-0.

## Introduction

In response to the increase of pregnant women who tested positive for SARS-CoV-2 presenting to birth hospitals for delivery within the setting of the COVID-19 pandemic in the spring of 2020, U.S. hospitals rapidly created and implemented clinical guidelines pertaining to maternal and newborn peripartum care. Large, population-level studies regarding risk of mother-to-infant transmission of SARS-CoV-2 and clinical manifestations of neonatal COVID-19 disease were lacking at this time, and are more recently emerging. In the context of this uncertainty, professional organizations issued different guidance in key areas of newborn care, which led to confusion and controversy [1, 2]. For example, on March 18, 2020, the *World Health Organization* (WHO) recommended that infants remain in close contact with SARS-CoV-2 positive mothers and directly breastfeed after delivery, if mothers are stable to do so [3], while the *American Academy of Pediatrics* (AAP) interim guidance from April 2, 2020 [4] and May 21, 2020 [5] recommended temporary separation of mother and infant as the safest course of action to minimize the risk of mother-to-infant transmission during the postpartum period. In comparison, guidance from the *Centers for Disease Control* (CDC) on April 4, 2020 [6] recommended that decisions about location of care “should be made on a case-by-case basis using shared decision-making between the mother and the clinical team.” The language was slightly modified on May 20, 2020 [7], to “risks and benefits of temporary separation of the mother and her baby should be discussed with the mother by the healthcare team, and decisions about temporary separation should be made in accordance with the mother’s wishes.”

The impact of these varying recommendations on clinical practice among U.S. birth hospitals is unknown. It is unclear whether hospitals may have preferentially adopted practices aligned with the WHO, AAP, CDC, other professional organizations [8], or other practices that were possibly tailored to their local context. Further, while extensive practice changes have undoubtedly occurred that focused on mother-infant dyads with positive SARS-CoV-2 testing, the extent to which hospitals may have changed practice for dyads *without* positive SARS-CoV-2 testing is unknown. Finally, whether hospital-level factors may have influenced what guidance a hospital adopted has not yet been examined.

In the context of these knowledge gaps, the primary purpose of our study was to examine variation in COVID-19 related obstetrical and newborn care practices among U.S. birth hospitals. Our secondary purpose was to examine variation in COVID-19 related obstetrical and newborn care practices according to hospital-level factors, including U.S. census region, highest level of neonatal care, and Baby-Friendly hospital status.

## Methods

### Population and setting

We ascertained COVID-19 newborn care practices at the hospital-level in a two-step process. First, we conducted a 5-7 minute, online, anonymous REDCap survey from May 26 to June 8, 2020 of *individual* physicians that provided newborn care at U.S. birth hospitals in the U.S. Because our goal was to distribute to a wide, national group of newborn care providers, we distributed the survey via email listservs of the AAP Sections on Hospital Medicine, Breastfeeding Medicine, and Neonatal Perinatal Medicine, as well as the Neonatal Physician Mothers Facebook group and on the Twitter feeds of the authors. We asked physicians to provide responses regarding hospital-level newborn care practices and guidelines during the week of May 17 to 24, 2020 at the hospital they “work at the most.” Second, we sorted the responses among individuals by the self-reported *U.S. birth hospital* that they work at most. If there was more than one response per hospital and the responses were the same, those responses constituted the responses for that hospital. If there was more than one response per hospital and the responses varied, we ascertained the name(s) and email address (es) of the newborn or neonatology physician medical director(s) at that hospital through personal email contacts of the authors, internet searches, and phone calls to hospitals, and clarified any discrepant responses. Specifically, we presented the survey question and answers verbatim along with the discrepant responses and asked for clarification regarding the most accurate response pertaining to their hospital’s approach of care from May 17 to 24, 2020. We incorporated the final “reconciled” responses by medical directors in the final data set of newborn care practices at the hospital-level. We chose this two-step, pragmatic approach to ascertain national COVID-19 newborn care practices to necessitate rapid feedback. This study was approved as an exempt study by the Boston University Medical Campus Institutional Review Board.

### Hospital Care Practices

We chose to examine obstetric and newborn care practices that were included in professional guidance by the WHO, AAP, and CDC, as well as topics that frequently arose in the Massachusetts statewide perinatal COVID-19 webinar series [9] and other similar national webinars [10]. We adapted survey questions from two previous Massachusetts statewide COVID-19 newborn care practice surveys administered to >20 hospital teams in April and May 2020 [9]. Our survey is included as an Appendix. We asked physicians to respond regarding hospital-level practices of interest, including testing of mothers, infants, and support persons, visitation, personal protective equipment (PPE) at delivery, location of care (separate rooms vs. rooming-in), skin-to-skin care in the first hour after birth, early baths, delayed cord clamping, direct breastfeeding, and discharge processes and timing.

### Hospital Characteristics

We examined responses by US region according to the US Census tract (Northeast, Midwest, South, and West) and highest level of neonatal care provided at the hospital (1, 2, 3, or 4) according to the AAP Guidelines for Perinatal Care [11]. We included definitions of levels of neonatal care in the survey for reference. We also examined responses according to the hospital’s Baby-Friendly status, which was obtained from the USA Baby-Friendly website [12].

### Individual Preference for Location of Care

In addition to hospital-level practices, we also examined individuals’ own preferred approach for location of care because this was a controversial practice at the time of survey distribution and professional organization guidance on this practice varied. We categorized responses as preferences for separation, rooming-in with precautions, or rooming-in without precautions; shared decision making on a case-by-case basis; no particular opinion; and other.

### Statistical Analysis

We examined prevalence and 95% confidence intervals (CIs) of hospital practices in our overall sample and then compared by US region, highest level of neonatal care, and Baby-Friendly hospital status, using chi-square tests. We then examined the extent to which individuals’ personal preferences for location of care agreed with their hospital’s recommended practice, using chi-square tests. All analyses were conducted using SAS 9.4.

## Results

**Fig. 1:**
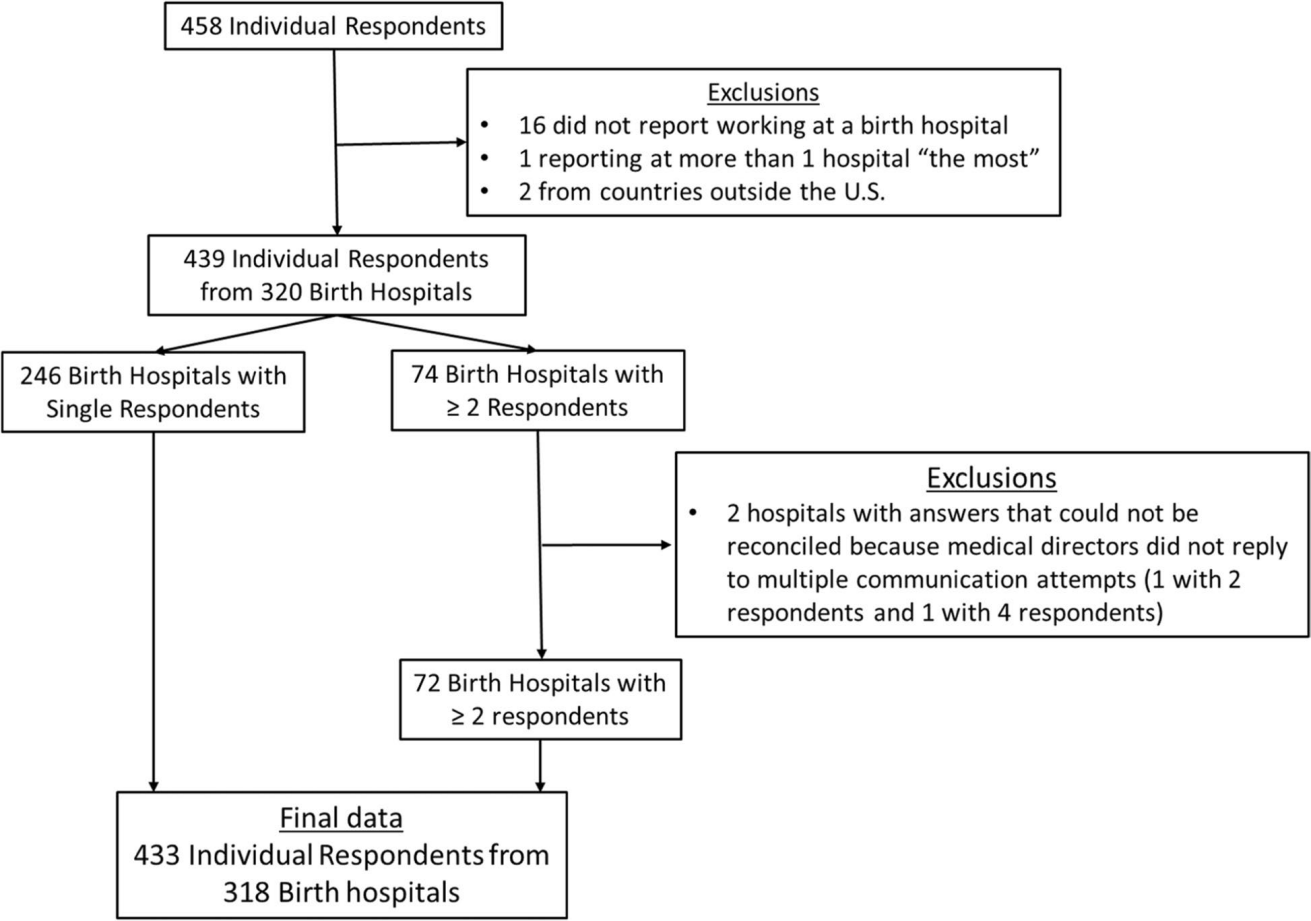
Participant Flow Diagram

A flow chart of participant and hospital inclusion criteria is shown in Figure 1. Among 458 total individual respondents, we excluded 16 that did not report working at a birth hospital, 1 that reported more than one birth hospital that they worked at “the most,” and 2 from non-U.S. states, leaving 439 individual respondents representing 320 unique birth hospitals in 46 states. Of these, 246 hospitals were represented by a single respondent, and 74 hospitals were represented by multiple respondents. The median and range of multiple respondents was 2 and 2 to 9, respectively. Among hospitals with multiple respondents, we excluded 2 due to lack of response from clinical/medical directors to reconcile discrepant answers. Our final sample consisted of 318 birth hospitals, comprised of 433 individual respondents for analysis. This represents approximately 11% of current US birthing hospitals.

Overall distribution of COVID-19 related hospital care practices are shown in Table 1. Nationally, 74% of hospitals offered universal testing to pregnant women who were anticipated to deliver. More than 95% of hospitals reported that the pediatric team wore N95 masks, eye protection, gloves and gowns as PPE for vaginal deliveries of COVID-19 positive women. Visitors were restricted to only 1 support person among 98% of hospitals and 78% did not offer or require any testing of support persons. Approach to location of newborn care varied substantially; we found that shared decision making was the preferred approach among 50% of hospitals, separate rooms among 31%, and care provided in the same room among 17%. Other breastfeeding support practices, including skin-to-skin care, and approaches to direct breastfeeding and provision of expressed mother’s milk also showed substantial variation (Table 1). Delayed or timed cord clamping for COVID-19 positive mothers was the approach among 65% of hospitals, compared to 93% for non-COVID-19 positive mothers; early bathing of infants born to COVID-19 positive mothers was the approach for 82% of hospitals, compared to 8% for infants born to non-COVID-19 positive mothers. With respect to infant testing, 53% of hospitals performed two tests and 29% performed one test, while 10% did not routinely test infants. We found that hearing screening was deferred until after discharge among 12% and circumcisions were deferred until after discharge among 15% of U.S. hospitals among infants born to mothers who tested positive for SARS-CoV-2, Finally, discharges occurred early for at least some presumed uninfected dyads among 59% of U.S. hospitals in our survey.

**Table 1:** COVID-19 Related Hospital Care Practices Among Mothers with Healthy, Term Infants Among US Birth Hospitals

Overall, n (%)	N (%)	95% CI
	318 (100%)	
**Obstetric and Delivery Practices**		
**Testing of Women Anticipated to Deliver**		
Universal testing	235 (73.9%)	68.7%, 78.6%
Testing based on signs and symptoms	68 (21.4%)	17.0%, 26.3%
Testing not routinely available for pregnant women	3 (0.9%)	0.2%, 2.7%
Other^1^	10 (3.1%)	1.5%, 5.7%
**PPE for COVID-19 positive women delivering vaginally (check all that apply)**		
N95	305 (95.9%)	93.1%, 97.8%
Regular Surgical Mask	17 (5.3%)	3.1%, 8.4%
Eye protection	310 (97.5%)	95.1%, 98.9%
Cap	206 (64.8%)	59.3%, 70.0%
Gown	305 (95.9%)	93.1%, 97.8%
Gloves	310 (97.5%)	95.1%, 98.9%
**Support persons for pregnant women on Labor and Delivery**		
No support persons	2 (0.6%)	0.1%, 2.3%
Only 1 support person	311 (97.8%)	95.5%, 99.1%
2 or more support persons	3 (0.9%)	0.2%, 2.7%
**Approach to testing asymptomatic support persons of COVID positive mothers**		
Not offered or required	250 (78.6%)	73.7%, 83.0%
Offer some testing	41 (12.9%)	9.4%, 17.1%
Required	24 (7.5%)	4.9%, 11.0%
**Newborn Care Practices**		
**Location of newborn care with COVID-19 Positive Mother**		
Separate room from mother	97 (30.5%)	25.5%, 35.9%
Same room as mother with precautions to maintain separation	55 (17.3%)	13.3%, 21.9%
Same room as mother with no precautions	1 (0.3%)	0.0%, 1.7%
Decisions based on shared decision making on a case-by-case basis	161 (50.6%)	45.0%, 56.3%
Other^2^	3 (0.9%)	0.2%, 2.7%
**Skin-to-skin care in first hour after birth with COVID-19 Positive Mother**		
Prohibited	40 (12.6%)	9.1%, 16.7%
Discouraged	114 (35.8%)	30.6%, 41.4%
Encouraged with precautions	33 (10.4%)	7.3%, 14.3%
Encouraged with no precautions	2 (0.6%)	0.1%, 2.3%
Decisions based on shared decision making on a case-by-case basis	127 (39.9%)	34.5%, 45.6%
**Delayed or timed cord clamping with COVID-19 Positive Mother**		
Yes	207 (65.1%)	59.6%, 70.3%
No	111 (34.9%)	29.7%, 40.4%
**Delayed or timed cord clamping with non-COVID Positive Mother**		
Yes	296 (93.1%)	89.7%, 95.6%
No	22 (6.9%)	4.4%, 10.3%
**Early baths (<4 hours) with COVID-19 Positive Mother**		
Yes	260 (81.8%)	77.1%, 85.8%
No	58 (18.2%)	14.2%, 22.9%
**Early baths (<4 hours) with non-COVID-19 Positive Mother**		
Yes	25 (7.9%)	5.2%, 11.4%
No	293 (92.1%)	88.6%, 94.8%
**Approach to direct breastfeeding with COVID-19 Positive Mother**		
Prohibited	15 (4.7%)	2.7%, 7.7%
Discouraged, but permitted if family strongly desires	101 (31.8%)	26.7%, 37.2%
Encouraged with precautions	49 (15.4%)	11.6%, 19.9%
Decisions based on shared decision making on a case-by-case basis	153 (48.1%)	42.5%, 53.8%
**Approach to expressed breast milk with COVID-19 Positive Mother (check all that apply)**		
May be given by the mother with precautions	189 (59.4%)	53.9%, 64.9%
May be given by another caregiver	245 (77.0%)	72.0%, 81.6%
Discouraged	3 (0.9%)	0.2%, 2.7%
**Approach to testing for an infant delivered by cesarean section with anticipated discharge on day 3 or 4 with a COVID-19 Positive Mother**		
We generally do not test infants	31 (9.7%)	6.7%, 13.6%
We do 1 test	93 (29.2%)	24.3%, 34.6%
We do 2 tests	169 (53.1%)	47.5%, 58.7%
More than 2 tests	3 (0.9%)	0.2%, 2.7%
Unsure	19 (6.0%)	3.6%, 9.2%
Other^3^	3 (0.9%)	0.2%, 2.7%
**Time of first test (*n*** **= 265; respondents that reported 1, 2, or 2+ tests)**		
Before 24 hours	12 (4.5%)	2.4%, 7.8%
Around 24 hours	199 (75.1%)	69.4%, 80.2%
Between 24-48 hours	42 (15.8%)	11.7%, 20.8%
Around 48 hours or after 48 hours	9 (3.4%)	1.6%, 6.3%
**Time of second test (*n*** **= 172; respondents that reported 2 or 2+ tests)**		
Between 24-48 hours	7 (4.1%)	1.7%, 8.2%
Around 48 hours	141 (82.0%)	75.4%, 87.4%
After 48 hours	23 (13.4%)	8.7%, 19.4%
**Discharge Processes for Infants with COVID-19 Positive Mothers**		
**Hearing screening**		
Have not changed process	244 (76.7%)	71.7%, 81.3%
Changed process, but occurs during hospitalization	32 (10.1%)	7.0%, 13.9%
Deferred until after discharge	39 (12.3%)	8.9%, 16.4%
**Circumcisions**		
Have not changed process	209 (65.7%)	60.2%, 70.9%
Changed process, but occurs during hospitalization	53 (16.7%)	12.7%, 21.2%
Deferred until after discharge	49 (15.4%)	11.6%, 19.9%
**Hepatitis B**		
Have not changed process	310 (97.5%)	95.1%, 98.9%
Changed process, but occurs during hospitalization	4 (1.3%)	0.3%, 3.2%
Deferred until after discharge	0 (0.0%)	--
**Bilirubin checks**		
Have not changed process	297 (93.4%)	90.1%, 95.9%
Changed process, but occurs during hospitalization	18 (5.7%)	3.4%, 8.8%
Deferred until after discharge	0 (0.0%)	--
**Newborn screen**		
Have not changed process	297 (93.4%)	90.1%, 95.9%
Changed process, but occurs during hospitalization	18 (5.7%)	3.4%, 8.8%
Deferred until after discharge	0 (0.0%)	--
**Congenital heart disease screen**		
Have not changed process	301 (94.7%)	91.6%, 96.9%
Changed process, but occurs during hospitalization	13 (4.1%)	2.2%, 6.9%
Deferred until after discharge	0 (0.0%)	--
**Red reflex**		
Have not changed process	306 (96.2%)	93.5%, 98.0%
Changed process, but occurs during hospitalization	5 (1.6%)	0.5%, 3.6%
Deferred until after discharge	3 (0.9%)	0.2%, 2.7%
**Discharge Processes for non-COVID-19 Positive Mother-Infant Dyads**		
**Timing of Discharge**		
Timing hasn’t really changed	129 (40.6%)	35.1%, 46.2%
Some dyads are discharged early	91 (28.6%)	23.7%, 33.9%
Many dyads are discharged early	73 (23.0%)	18.4%, 28.0%
All dyads discharge early unless a medical contraindication	24 (7.5%)	4.9%, 11.0%

*PPE* personal protective equipment, *L&D* labor and delivery

^1^Other maternal testing answers were test based on symptoms OR a scheduled admission for delivery (*n* = 4), test for a scheduled admission (*n* = 4), “based on obstetrical practice” (*n* = 1), and “universal test at 38 weeks” (*n* = 1)

^2^Other location of newborn care answers were if mother asymptomatic and desires same room and if mother symptomatic separate rooms (*n* = 3)

^3^Other newborn testing answers were “test infant if symptomatic” (*n* = 1), decision made on case by case basis by provider (*n* = 1) or infection control (*n* = 1)

We found substantial regional variation in several COVID-19 related hospital practices (Table 2). The likelihood that a hospital offered universal testing of women expected to deliver and offering or requiring testing for asymptomatic support persons was highest in the Northeast. Regarding infant testing, hospitals in the South had the highest rate of testing each infant twice. Care in separate rooms and prohibiting and discouraging direct breastfeeding were highest in the South, whereas shared decision making was used most often among hospitals in the West. Early discharge of dyads was reported most frequently among hospitals in the Northeast.

**Table 2:** COVID-19 Related Hospital Care Practices Among Mothers with Healthy, Term Infants According to US Region^1, 2^

Overall, n (%)	Northeast	Midwest	South	West	***p***-value
**Obstetric and Delivery Practices**					
**Testing of Women Anticipated to Deliver**					0.043
Universal testing	69 (88.5%)	51 (77.3%)	64 (67.4%)	51 (64.6%)	
Testing based on signs and symptoms	7 (9.0%)	10 (15.2%)	27 (28.4%)	24 (30.4%)	
Testing not routinely available for pregnant women	1 (1.3%)	1 (1.5%)	0 (0.0%)	1 (1.3%)	
Other^3^	1 (1.3%)	4 (6.1%)	3 (3.2%)	2 (2.5%)	
**PPE for COVID-19 positive women delivering vaginally (check all that apply)**					
N95	76 (97.4%)	61 (92.4%)	92 (96.8%)	76 (96.2%)	0.434
Regular Surgical Mask	3 (3.8%)	6 (9.1%)	3 (3.2%)	5 (6.3%)	0.358
Eye protection	76 (97.4%)	65 (98.5%)	92 (96.8%)	77 (97.5%)	0.934
Cap	60 (76.9%)	36 (54.5%)	69 (72.6%)	41 (51.9%)	<.001
Gown	77 (98.7%)	63 (95.5%)	93 (97.9%)	72 (91.1%)	0.068
Gloves	78 (100.0%)	64 (97.0%)	94 (98.9%)	74 (93.7%)	0.055
**Support persons for pregnant women on Labor and Delivery**					0.429
No support persons	0 (0.0%)	1 (1.5%)	1 (1.1%)	0 (0.0%)	
Only 1 support person	77 (98.7%)	62 (93.9%)	93 (97.9%)	79 (100.0%)	
2 or more support persons	1 (1.3%)	2 (3.0%)	0 (0.0%)	0 (0.0%)	
**Approach to testing asymptomatic support persons of COVID positive mothers**					0.006
Not offered or required	52 (66.7%)	58 (87.9%)	77 (81.1%)	63 (79.7%)	
Offer some testing	12 (15.4%)	6 (9.1%)	10 (10.5%)	13 (16.5%)	
Required	14 (17.9%)	2 (3.0%)	6 (6.3%)	2 (2.5%)	
**Newborn Care Practices**					
**Location of newborn care with COVID-19 Positive Mother**					0.057
Separate room from mother	17 (21.8%)	22 (33.3%)	40 (42.1%)	18 (22.8%)	
Same room as mother with precautions to maintain separation	20 (25.6%)	10 (15.2%)	13 (13.7%)	12 (15.2%)	
Same room as mother with no precautions	1 (1.3%)	0 (0.0%)	0 (0.0%)	0 (0.0%)	
Decisions based on shared decision making on a case-by-case basis	38 (48.7%)	34 (51.5%)	40 (42.1%)	49 (62.0%)	
Other^4^	2 (2.6%)	0 (0.0%)	1 (1.1%)	0 (0.0%)	
**Skin-to-skin care in first hour after birth with COVID-19 Positive Mother**					0.169
Prohibited	10 (12.8%)	8 (12.1%)	17 (17.9%)	5 (6.3%)	
Discouraged	24 (30.8%)	29 (43.9%)	34 (35.8%)	27 (34.2%)	
Encouraged with precautions	10 (12.8%)	6 (9.1%)	9 (9.5%)	8 (10.1%)	
Encouraged with no precautions	0 (0.0%)	0 (0.0%)	2 (2.1%)	0 (0.0%)	
Decisions based on shared decision making on a case-by-case basis	34 (43.6%)	23 (34.8%)	33 (34.7%)	37 (46.8%)	
Other					
**Delayed or timed cord clamping with COVID-19 Positive Mother**					0.087
Yes	57 (73.1%)	39 (59.1%)	55 (57.9%)	56 (70.9%)	
No	21 (26.9%)	27 (40.9%)	40 (42.1%)	23 (29.1%)	
**Delayed or timed cord clamping with non-COVID Positive Mother**					0.066
Yes	73 (93.6%)	61 (92.4%)	84 (88.4%)	78 (98.7%)	
No	5 (6.4%)	5 (7.6%)	11 (11.6%)	1 (1.3%)	
**Early baths (<4 hours) with COVID-19 Positive Mother**					0.152
Yes	60 (76.9%)	59 (89.4%)	80 (84.2%)	61 (77.2%)	
No	18 (23.1%)	7 (10.6%)	15 (15.8%)	18 (22.8%)	
**Early baths (<4 hours) with non-COVID-19 Positive Mother**					0.616
Yes	6 (7.7%)	5 (7.6%)	10 (10.5%)	4 (5.1%)	
No	72 (92.3%)	61 (92.4%)	85 (89.5%)	75 (94.9%)	
**Approach to direct breastfeeding with COVID-19 Positive Mother**					0.028
Prohibited	3 (3.8%)	4 (6.1%)	6 (6.3%)	2 (2.5%)	
Discouraged, but permitted if family strongly desires	15 (19.2%)	22 (33.3%)	43 (45.3%)	21 (26.6%)	
Encouraged with precautions	15 (19.2%)	8 (12.1%)	11 (11.6%)	15 (19.0%)	
Decisions based on shared decision making on a case-by-case basis	45 (57.7%)	32 (48.5%)	35 (36.8%)	41 (51.9%)	
**Approach to expressed breast milk with COVID-19 Positive Mother (check all that apply)**					
May be given by the mother with precautions	48 (61.5%)	39 (59.1%)	52 (54.7%)	50 (63.3%)	0.682
May be given by another caregiver	59 (75.6%)	56 (84.8%)	72 (75.8%)	58 (73.4%)	0.387
Discouraged	0 (0.0%)	1 (1.5%)	0 (0.0%)	2 (2.5%)	0.260
**Approach to testing for an infant delivered by cesarean section with anticipated discharge on day 3 or 4 with COVID-19 Positive Mother**					0.003
We generally do not test infants	6 (7.7%)	8 (12.1%)	6 (6.3%)	11 (13.9%)	
We do 1 test	35 (44.9%)	15 (22.7%)	20 (21.1%)	23 (29.1%)	
We do 2 tests	35 (44.9%)	37 (56.1%)	63 (66.3%)	34 (43.0%)	
More than 2 tests	0 (0.0%)	1 (1.5%)	1 (1.1%)	1 (1.3%)	
Unsure	0 (0.0%)	5 (7.6%)	4 (4.2%)	10 (12.7%)	
Other^5^	2 (2.6%)	0 (0.0%)	1 (1.1%)	0 (0.0%)	
**Time of first test (*n*** **= 262; respondents that reported 1, 2, or 2+ tests)**					0.041
Before 24 hours	1 (1.4%)	1 (1.9%)	5 (6.0%)	5 (8.6%)	
Around 24 hours	49 (70.0%)	37 (69.8%)	69 (82.1%)	44 (75.9%)	
Between 24-48 hours	17 (24.3%)	10 (18.9%)	7 (8.3%)	8 (13.8%)	
Around 48 hours or after 48 hours	2 (2.8%)	5 (9.4%)	2 (2.4%)	0 (0.0%)	
**Time of second test (*n*** **= 171; respondents that reported 2 or 2+ tests)**					0.341
Between 24-48 hours	1 (2.9%)	0 (0.0%)	4 (6.3%)	2 (5.7%)	
Around 48 hours	29 (82.9%)	29 (76.3%)	55 (85.9%)	28 (80.0%)	
After 48 hours	5 (14.3%)	9 (23.7%)	4 (6.3%)	5 (14.3%)	
**Discharge Processes**					
**Hearing screening**					0.131
Have not changed process	59 (75.6%)	51 (77.3%)	73 (76.8%)	61 (77.2%)	
Changed process, but occurs during hospitalization	11 (14.1%)	10 (15.2%)	4 (4.2%)	7 (8.9%)	
Deferred until after discharge	8 (10.3%)	5 (7.6%)	17 (17.9%)	9 (11.4%)	
**Circumcisions**					0.107
Have not changed process	50 (64.1%)	43 (65.2%)	59 (62.1%)	57 (72.2%)	
Changed process, but occurs during hospitalization	17 (21.8%)	16 (24.2%)	12 (12.6%)	8 (10.1%)	
Deferred until after discharge	10 (12.8%)	7 (10.6%)	21 (22.1%)	11 (13.9%)	
**Hepatitis B**					0.396
Have not changed process	76 (97.4%)	65 (98.5%)	94 (98.9%)	75 (94.9%)	
Changed process, but occurs during hospitalization	2 (2.6%)	0 (0.0%)	0 (0.0%)	2 (2.5%)	
Deferred until after discharge	0 (0.0%)	0 (0.0%)	0 (0.0%)	0 (0.0%)	
**Bilirubin checks**					0.007
Have not changed process	67 (85.9%)	63 (95.5%)	91 (95.8%)	76 (96.2%)	
Changed process, but occurs during hospitalization	11 (14.1%)	3 (4.5%)	3 (3.2%)	1 (1.3%)	
Deferred until after discharge	0 (0.0%)	0 (0.0%)	0 (0.0%)	0 (0.0%)	
**Newborn screen**					0.231
Have not changed process	70 (89.7%)	63 (95.5%)	89 (93.7%)	75 (94.9%)	
Changed process, but occurs during hospitalization	8 (10.3%)	3 (4.5%)	5 (5.3%)	2 (2.5%)	
Deferred until after discharge	0 (0.0%)	0 (0.0%)	0 (0.0%)	0 (0.0%)	
**Congenital heart disease screen**					0.450
Have not changed process	72 (92.3%)	63 (95.5%)	92 (96.8%)	74 (93.7%)	
Changed process, but occurs during hospitalization	6 (7.7%)	2 (3.0%)	2 (2.1%)	3 (3.8%)	
Deferred until after discharge	0 (0.0%)	0 (0.0%)	0 (0.0%)	0 (0.0%)	
**Red reflex**					0.323
Have not changed process	74 (94.9%)	65 (98.5%)	91 (95.8%)	76 (96.2%)	
Changed process, but occurs during hospitalization	3 (3.8%)	1 (1.5%)	0 (0.0%)	1 (1.3%)	
Deferred until after discharge	1 (1.3%)	0 (0.0%)	2 (2.1%)	0 (0.0%)	
**Discharge Processes for non-COVID-19 Positive Mother-Infant Dyads**					
**Timing of Discharge**					<0.001
Timing hasn’t really changed	17 (21.8%)	28 (42.4%)	48 (50.5%)	36 (45.6%)	
Some dyads are discharged early	20 (25.6%)	16 (24.2%)	29 (30.5%)	26 (32.9%)	
Many dyads are discharged early	29 (37.2%)	20 (30.3%)	16 (16.8%)	8 (10.1%)	
All dyads discharge early unless a medical contraindication	12 (15.4%)	2 (3.0%)	2 (2.1%)	8 (10.1%)	

*PPE* personal protective equipment, *L&D* labor and delivery

^1^Chi-square *p*-values shown; Missing answers not shown (this occurred <1.5% of all questions)

^2^Northeast includes Maine, New Hampshire, Vermont, Massachusetts, Connecticut, Rhode Island, New York, New Jersey, Pennsylvania, Delaware, Maryland, Virginia, West Virginia, and Washington D.C.; Midwest includes Minnesota, Wisconsin, Michigan, Iowa, Illinois, Missouri, Indiana, Ohio, and Kentucky; South includes Arkansas, Tennessee, North Carolina, Louisiana, Mississippi, Alabama, Georgia, and Florida; West includes Washington, Oregon, Idaho, California, Nevada, Utah, Arizona, Hawaii, and Alaska

^3^Other maternal testing answers were test based on symptoms OR a scheduled admission for delivery (*n* = 4), test for a scheduled admission (*n* = 4), “based on obstetrical practice” (*n* = 1), and “universal test at 38 weeks” (*n* = 1)

^4^Other location of newborn care answers were if mother asymptomatic and desires same room and if mother symptomatic separate rooms (*n* = 3)

^5^Other newborn testing answers were “test infant if symptomatic” (*n* = 1), decision made on case by case basis by provider (*n* = 1) or infection control (*n* = 1)

Hospital practices did not vary significantly according to highest level of neonatal care (Additional File A), with the exception of skin-to-skin care in the first hour after birth among mothers with positive tests, where prohibiting or discouraging skin-to-skin care was highest among hospitals providing level 3 and 4 neonatal care (51%), compared to hospitals with level 1 (44%) and level 2 (40%) neonatal care (*p* = 0.024). Hospital breastfeeding support practices did not vary by Baby-Friendly hospital status (Additional File B).

Finally, among all individual respondents, we considered each individual’s personal preferred approach to location of care, and whether this differed from their hospital’s approach. Among 433 individual respondents, 174 (40%) preferred that decisions be based on shared decision making on a case-by-case basis, 128 (30%) preferred care of dyads in same room with precautions, 105 (24%) preferred care of dyads in separate rooms, 14 (3%) had no particular opinion, 8 (2%) preferred care of dyads in the same room without precautions, and 4 (0.9%) gave other responses. Table 3 shows the concordance of hospital and individual preferred approaches to location of care, restricted to the 403 respondents (93% of sample) that preferred either shared decision making, same room with precautions, or separate rooms and reported that their hospitals used one of these three approaches. Among hospitals with a separation approach, 51% of respondents from those hospitals preferred an alternative approach. Among hospitals that used a same room with precautions approach, 36% of respondents from those hospitals preferred an alternative approach, and among hospitals that used a shared-decision making approach, 43% of respondents from those hospitals preferred an alternative approach.

**Table 3:** Congruence Between Personal Preference and Hospital Approach to Location of Care^1^

Hospital Approach to Location of Care	Personal Preferred Approach to Location of Care^**2**^			Total (sum of row%)
	Separate rooms N (row%)	Same room with some precautions N (row%)	Shared decision making on a case-by-case basis N (row%)	
Separate rooms	**65 (48.9%)**	30 (22.6%)	38 (28.6%)	133 (33.0%)
Same room with precautions	4 (5.3%)	**48 (64.0%)**	23 (30.7%)	75 (18.6%)
Shared decision making on a case-by-case basis	36 (18.5%)	48 (24.6%)	**111 (56.9%)**	195 (48.4%)
Total (column %)	105 (26.1%)	126 (31.3%)	172 (42.5%)	403 (100%)

^1^ Includes 403 individual responses. We excluded 30 respondents (7%) with: A) no particular personal opinion (*n* = 14), personal preference was same room with no precautions (*n* = 8), personal preference was “other” (*n* = 4), hospital approach was same room with no precautions (*n* = 1), and hospital approach was “other” (*n* = 3).

## Discussion

Physicians from a large sample of hospitals across the U.S. surveyed regarding COVID-19 related newborn care practices from May 17 to 24th demonstrated great variation in several breastfeeding support practices, including location of newborn care, direct breastfeeding, and skin-to-skin care after birth. Adoption of a “shared decision making” approach was most common in the areas of highest variation. We also found that nearly 60% of hospitals discharged at least some of their presumed uninfected dyads earlier than their usual practice prior to the pandemic. Finally, SARS-CoV-2 testing of mothers, asymptomatic support persons, and infants, as well as approaches to location of care, direct breastfeeding and timing of discharge varied according to US region, but not by highest level of neonatal care or Baby-Friendly Hospital Status.

Hospitals routinely create guidelines or policies with the goal of reducing provider variation and/or optimizing the delivery of standardized, evidenced-based care. Considering the paucity of population-level data that could best inform practices for newborn care in the setting of the COVID-19 pandemic in the spring of 2020, it is not surprising that we found wide national variation in several COVID-19 newborn care practices. Similar to our findings, Ahmad et al recently reported variation in maternal testing, location of care, and direct breastfeeding among 368 hospitals assessed by 4 serial surveys from March to August 2020 among U.S. hospitals affiliated with the Mednax network [13]. While we did not perform serial surveys to examine changes over time, our study included nearly all US states, therefore representing a broader range of US hospitals and we additionally examined newborn testing and discharge practices, skin-to-skin care, delayed cord clamping and early baths. Variation was likely driven in part by differing guidance by major professional organizations in the spring of 2020. Our study showed that among several breastfeeding support practices, shared decision making, which was endorsed by the CDC in their statement on April 4, 2020 [6], was the most frequently used hospital approach and most frequent personal preferred approach to location of care among individual survey respondents. Shared decision making occurs when healthcare professionals and patients make decisions together with both parties cognizant of available evidence, the limits of evidence, and uncertainties related to the possible benefits and harms of each options [14]. We speculate that, in the setting of uncertainty regarding the health risks and benefits of these various practices, many hospital teams and individual providers felt comfortable engaging families directly in the process of decision-making.

We also found that some hospital practices were not consistent with professional organization guidelines. Local context may be a driver for this variation. For example, we found that universal testing of women anticipated to deliver was most frequent in Northeastern hospitals, which may have represented increased local testing availability, and the fact that the pandemic was most acute in this region at the time of survey administration. It is also possible that changes in discharge processes, such as timing of circumcisions and hearing screenings, may have been driven by local preferences of hospital personnel or by a need to conserve PPE. Indeed, an audit completed by 359 hospitals conducted by the Vermont Oxford Network in April 2020 revealed shortages in COVID-19 testing and PPE [15].

While some variation based on local context is likely desirable, other types of variation may not be. If differences in hospital practices reflect external circumstances, rather than local hospital factors, variations may lead to inequities in care. For example, we found higher proportions of hospitals prohibiting/discouraging direct breastfeeding and separating mothers and newborns, practices known to negatively impact the establishment of breastfeeding [16], in the South, compared to other U.S. regions. Prior to the pandemic, breastfeeding rates were lowest in the South [17]. Thus, it is possible that adoption of certain COVID-19 related hospital practices may have exacerbated existing regional disparities in breastfeeding.

The majority of hospitals in our sample (59%) discharged at least some of their presumed uninfected dyads early, a practice that was not recommended by the WHO, AAP, or the CDC. This may be concerning because many important processes occur during the postpartum hospitalization for the mother and infant which may be missed with early discharge. For example, postpartum women are followed for hypertension, bleeding, and wound healing [11]. Newborns are followed for jaundice, weight loss, and sepsis and require a variety of screening procedures [11]. Further, for most dyads, two days or more are needed for the medical team to assess the adequacy of breastfeeding and for trained professionals to assist with breastfeeding [11, 16]. Early discharge puts mother-infant dyads at risk for a variety of medical complications that may be less well-monitored in the outpatient setting. In addition, access to outpatient medical providers and lactation support was limited in many areas in the early stages of the COVID-19 pandemic, which may have put maternal-infant dyads at even higher risk due to the decreased ability to ensure good follow-up. Our study did not assess the reasons for early discharge. It is possible that families were asking for early discharge because of their concern for contracting COVID-19 in the hospital or their dissatisfaction with visitation restrictions or other hospital procedures. It is also possible that providers may have more readily offered early discharge because of an interest in reducing any unnecessary staff exposures to patients in the hospital setting. Further examination of the rationale for early discharge is needed to best inform public health messaging and avoid potential adverse consequences of early discharge of healthy mother-infant dyads.

We found that the number of support persons for laboring women was restricted to 1 among 98% of our sample. While the impact of this practice is unclear for laboring mothers, others have reported adverse effects on parents following widespread restrictions of visitors in the neonatal intensive care unit [18, 19]. Parents experienced negative impacts on bonding, receipt of provider information, and breastfeeding. Further investigation of the impact of COVID-19 restrictions on the number of support persons during labor and delivery and the postpartum hospitalization is needed.

The strengths of our study are inclusion of a large array of hospitals across 46 U.S. states and examination of many perinatal practices at the hospital-level. We also used a two-step approach to sample hospitals. We recognize that a survey administered directly to clinical leaders of newborn nurseries across the U.S. would have been preferred, however, we were unable to identify a comprehensive directory of this nature with up-to-date contact information. It is possible that individual respondents may not have had the most up-to-date and accurate information regarding their hospital’s current practices. We minimized this possibility by administering the survey over a short time frame and specifically including the dates during which we aimed to assess current hospital practices in our prompt. It is possible that respondents may have reported their personal practices, rather than the hospital’s current practice. Additionally, individuals that completed our survey via listservs and social media may not represent individuals at all US hospitals. There could be bias in response based on individuals particularly interested in the topic. Further, not all physicians working in birthing facilities are members of the AAP Sections of Neonatal Perinatal Medicine, Breastfeeding, and Hospital Medicine or the Neonatal Physician Mother Facebook group. Finally, due to the rapidly evolving state of the COVID-19 pandemic, the findings of our survey may not reflect current hospital practices implemented after professional organizations published guidelines after June 2020 [20, 21]. Nonetheless, the implications of hospital practice variation (overall and by region), including the importance of local context, will likely continue to inform on-going determinations of optimal perinatal care approaches.

## Conclusion

Variation in COVID-19 newborn care practices among U.S. hospitals suggests that mother-infant dyads received different care depending on the hospital and region where delivery took place in late spring 2020. As more evidence and population-level data emerges to inform optimal COVID-19 newborn care practices, consistent adoption of these practices will promote health outcomes and reduce practice variation which may lead to improved health equity. The high rate of early discharge (nearly 60%) among mother-infant dyads presumed to be uninfected is worrisome in light of potential maternal and neonatal complications that may not be detected or treated timely in the outpatient setting. Overall, we believe that the first surge of the COVID-19 pandemic highlighted the need for professional organizations to work together when publishing guidance, development of data repositories to inform new evidence to drive decisions about best practice and leveraging existing regional and national networks of perinatal centers to facilitate rapid adoption of best practices as they emerge.

## Supplementary Information

**Additional file 1.**

**Additional file 2.**

## Data Availability

The data set used during the current study are available from the corresponding author on reasonable request

## Abbreviations

WHO: World Health Organization
AAP: American Academy of Pediatrics
CDC: Centers for Disease Control
US: United States
PPE: Personal protective equipment
CI: Confidence interval
