# Supplementary material for: Variation in United States COVID-19 newborn care practices: results of an online physician survey
Source: BMC Pediatr. 2022 Jan 21;22:55. doi: 10.1186/s12887-022-03129-0 (PMC8776982; doi:10.1186/s12887-022-03129-0)
Supplement: Supplementary file 1 — Additional file 1. [file 12887_2022_3129_MOESM1_ESM.docx]

**Additional File.**

Variation in United States COVID-19 Newborn Care Practices: Results of an Online Physician Survey

Margaret G. Parker, MD, MPH,^1^ Arun Gupta, MD,^2^ Helen Healy, MD, MPH^3^ Aviel Peaceman, MPH,^1^ Stephen M. Kerr, MPH,^4^ Timothy C. Heeren, PhD,^5^ Mark L Hudak, MD,^6^ Munish Gupta, MD, MMSc^7^

**Additional File A. COVID-19 Related Hospital Care Practices Among Mothers with Healthy, Term Infants According to Highest Level of Neonatal Care^1^**

|  | **Level 1** | **Level 2** | **Level 3/4** | **p-value** |
| --- | --- | --- | --- | --- |
| Overall, n (%) | 27 (8.5%) | 62 (19.5%) | 229 (72.0%) |  |
| **Obstetric and Delivery Practices** |  |  |  |  |
| **Testing of Women Anticipated to Deliver** |  |  |  | 0.238 |
| Universal testing | 20 (74.1%) | 43 (69.4%) | 172 (75.1%) |  |
| Testing based on signs and symptoms | 6 (22.2%) | 13 (21.0%) | 49 (21.4%) |  |
| Testing not routinely available for pregnant women | 1 (3.7%) | 1 (1.6%) | 1 (0.4%) |  |
| Other^2^ | 0 (0.0%) | 5 (8.1%) | 5 (2.2%) |  |
| **PPE for COVID-19 positive women delivering vaginally (check all that apply)** |  |  |  |  |
| N95 | 25 (92.6%) | 59 (95.2%) | 221 (96.5%) | 0.590 |
| Regular Surgical Mask | 2 (7.4%) | 3 (4.8%) | 12 (5.2%) | 0.877 |
| Eye protection | 25 (92.6%) | 59 (95.2%) | 226 (98.7%) | 0.069 |
| Cap | 17 (63.0%) | 39 (62.9%) | 150 (65.5%) | 0.911 |
| Gown | 26 (96.3%) | 57 (91.9%) | 222 (96.9%) | 0.209 |
| Gloves | 27 (100.0%) | 59 (95.2%) | 224 (97.8%) | 0.339 |
| **Support persons for pregnant women on Labor and Delivery** |  |  |  | 0.116 |
| No support persons | 0 (0.0%) | 1 (1.6%) | 1 (0.4%) |  |
| Only 1 support person | 26 (96.3%) | 59 (95.2%) | 226 (98.7%) |  |
| 2 or more support persons | 0 (0.0%) | 2 (3.2%) | 1 (0.4%) |  |
| **Approach to testing asymptomatic support persons of COVID positive mothers** |  |  |  | 0.678 |
| Not offered or required | 21 (77.8%) | 50 (80.6%) | 179 (78.2%) |  |
| Offer some testing | 2 (7.4%) | 8 (12.9%) | 31 (13.5%) |  |
| Required | 4 (14.8%) | 4 (6.5%) | 16 (7.0%) |  |
| **Newborn Care Practices** |  |  |  |  |
| **Location of newborn care with COVID-19 Positive Mother** |  |  |  | 0.446 |
| Separate room from mother | 4 (14.8%) | 17 (27.4%) | 76 (33.2%) |  |
| Same room as mother with precautions to maintain separation | 9 (33.3%) | 12 (19.4%) | 34 (14.8%) |  |
| Same room as mother with no precautions | 0 (0.0%) | 0 (0.0%) | 1 (0.4%) |  |
| Decisions based on shared decision making on a case-by-case basis | 14 (51.9%) | 33 (53.2%) | 114 (49.8%) |  |
| Other^3^ | 0 (0.0%) | 0 (0.0%) | 3 (1.3%) |  |
| **Skin-to-skin care in first hour after birth with COVID-19 Positive Mother** |  |  |  | 0.024 |
| Prohibited | 5 (18.5%) | 3 (4.8%) | 32 (14.0%) |  |
| Discouraged | 7 (25.9%) | 22 (35.5%) | 85 (37.1%) |  |
| Encouraged with precautions | 5 (18.5%) | 7 (11.3%) | 21 (9.2%) |  |
| Encouraged with no precautions | 1 (3.7%) | 0 (0.0%) | 1 (0.4%) |  |
| Decisions based on shared decision making on a case-by-case basis | 9 (33.3%) | 28 (45.2%) | 90 (39.3%) |  |
| **Delayed or timed cord clamping with COVID-19 Positive Mother** |  |  |  | 0.398 |
| Yes | 19 (70.4%) | 36 (58.1%) | 152 (66.4%) |  |
| No | 8 (29.6%) | 26 (41.9%) | 77 (33.6%) |  |
| **Delayed or timed cord clamping with non-COVID Positive Mother** |  |  |  | 0.329 |
| Yes | 27 (100.0%) | 57 (91.9%) | 212 (92.6%) |  |
| No | 0 (0.0%) | 5 (8.1%) | 17 (7.4%) |  |
| **Early baths (<4 hours) with COVID-19 Positive Mother** |  |  |  | 0.385 |
| Yes | 23 (85.2%) | 47 (75.8%) | 190 (83.0%) |  |
| No | 4 (14.8%) | 15 (24.2%) | 39 (17.0%) |  |
| **Early baths (<4 hours) with non-COVID-19 Positive Mother** |  |  |  | 0.994 |
| Yes | 2 (7.4%) | 5 (8.1%) | 18 (7.9%) |  |
| No | 25 (92.6%) | 57 (91.9%) | 211 (92.1%) |  |
| **Approach to direct breastfeeding with COVID-19 Positive Mother** |  |  |  | 0.737 |
| Prohibited | 1 (3.7%) | 1 (1.6%) | 13 (5.7%) |  |
| Discouraged, but permitted if family strongly desires | 7 (25.9%) | 19 (30.6%) | 75 (32.8%) |  |
| Encouraged with precautions | 6 (22.2%) | 11 (17.7%) | 32 (14.0%) |  |
| Decisions based on shared decision making on a case-by-case basis | 13 (48.1%) | 31 (50.0%) | 109 (47.6%) |  |
| **Approach to expressed breast milk with COVID-19 Positive Mother (check all that apply)** |  |  |  |  |
| May be given by the mother with precautions | 14 (51.9%) | 35 (56.5%) | 140 (61.1%) | 0.563 |
| May be given by another caregiver | 19 (70.4%) | 48 (77.4%) | 178 (77.7%) | 0.689 |
| Discouraged | 0 (0.0%) | 1 (1.6%) | 2 (0.9%) | 0.753 |
| **Approach to testing for an infant delivered by cesarean section with anticipated discharge on day 3 or 4 with COVID-19 Positive Mother** |  |  |  | 0.435 |
| We generally do not test infants | 5 (18.5%) | 3 (4.8%) | 23 (10.0%) |  |
| We do 1 test | 7 (25.9%) | 20 (32.3%) | 66 (28.8%) |  |
| We do 2 tests | 13 (48.1%) | 31 (50.0%) | 125 (54.6%) |  |
| More than 2 tests | 0 (0.0%) | 0 (0.0%) | 3 (1.3%) |  |
| Unsure | 2 (7.4%) | 7 (11.3%) | 10 (4.4%) |  |
| Other^4^ | 0 (0.0%) | 1 (1.6%) | 2 (0.9%) |  |
| **Time of first test (n = 262; respondents that reported 1, 2, or 2+ tests)** |  |  |  | 0.160 |
| Before 24 hours | 1 (5.0%) | 1 (2.0%) | 10 (5.2%) |  |
| Around 24 hours | 13 (65.0%) | 36 (70.6%) | 150 (77.3%) |  |
| Between 24-48 hours | 5 (25.0%) | 13 (25.5%) | 24 (12.4%) |  |
| Around 48 hours or after 48 hours | 1 (5.0%) | 1 (2.0%) | 7 (3.6%) |  |
| **Time of second test (n = 171; respondents that reported 2 or 2+ tests)** |  |  |  | 0.309 |
| Between 24-48 hours | 0 (0.0%) | 0 (0.0%) | 7 (5.5%) |  |
| Around 48 hours | 13 (100.0%) | 24 (77.4%) | 104 (81.3%) |  |
| After 48 hours | 0 (0.0%) | 7 (22.6%) | 16 (12.5%) |  |
| **Discharge Processes** |  |  |  |  |
| **Hearing screening** |  |  |  | 0.166 |
| Have not changed process | 22 (81.5%) | 47 (75.8%) | 175 (76.4%) |  |
| Changed process, but occurs during hospitalization | 3 (11.1%) | 9 (14.5%) | 20 (8.7%) |  |
| Deferred until after discharge | 2 (7.4%) | 4 (6.5%) | 33 (14.4%) |  |
| **Circumcisions** |  |  |  | 0.534 |
| Have not changed process | 16 (59.3%) | 38 (61.3%) | 155 (67.7%) |  |
| Changed process, but occurs during hospitalization | 5 (18.5%) | 15 (24.2%) | 33 (14.4%) |  |
| Deferred until after discharge | 5 (18.5%) | 7 (11.3%) | 37 (16.2%) |  |
| **Hepatitis B** |  |  |  | 0.271 |
| Have not changed process | 27 (100.0%) | 58 (93.5%) | 225 (98.3%) |  |
| Changed process, but occurs during hospitalization | 0 (0.0%) | 2 (3.2%) | 2 (0.9%) |  |
| Deferred until after discharge | 0 (0.0%) | 0 (0.0%) | 0 (0.0%) |  |
| **Bilirubin checks** |  |  |  | 0.322 |
| Have not changed process | 26 (96.3%) | 57 (91.9%) | 214 (93.4%) |  |
| Changed process, but occurs during hospitalization | 1 (3.7%) | 3 (4.8%) | 14 (6.1%) |  |
| Deferred until after discharge | 0 (0.0%) | 0 (0.0%) | 0 (0.0%) |  |
| **Newborn screen** |  |  |  | 0.323 |
| Have not changed process | 25 (92.6%) | 56 (90.3%) | 216 (94.3%) |  |
| Changed process, but occurs during hospitalization | 2 (7.4%) | 4 (6.5%) | 12 (5.2%) |  |
| Deferred until after discharge | 0 (0.0%) | 0 (0.0%) | 0 (0.0%) |  |
| **Congenital heart disease screen** |  |  |  | 0.034 |
| Have not changed process | 25 (92.6%) | 55 (88.7%) | 221 (96.5%) |  |
| Changed process, but occurs during hospitalization | 2 (7.4%) | 4 (6.5%) | 7 (3.1%) |  |
| Deferred until after discharge | 0 (0.0%) | 0 (0.0%) | 0 (0.0%) |  |
| **Red reflex** |  |  |  | 0.142 |
| Have not changed process | 27 (100.0%) | 58 (93.5%) | 221 (96.5%) |  |
| Changed process, but occurs during hospitalization | 0 (0.0%) | 1 (1.6%) | 4 (1.7%) |  |
| Deferred until after discharge | 0 (0.0%) | 0 (0.0%) | 3 (1.3%) |  |
| **Discharge Processes for non-COVID-19 Positive Mother-Infant Dyads** |  |  |  |  |
| **Timing of Discharge** |  |  |  | 0.451 |
| Timing hasn’t really changed | 9 (33.3%) | 21 (33.9%) | 99 (43.2%) |  |
| Some dyads are discharged early | 8 (29.6%) | 18 (29.0%) | 65 (28.4%) |  |
| Many dyads are discharged early | 8 (29.6%) | 18 (29.0%) | 47 (20.5%) |  |
| All dyads discharge early unless a medical contraindication | 2 (7.4%) | 4 (6.5%) | 18 (7.9%) |  |

PPE = personal protective equipment; L&D = labor and delivery

^1^Chi-square p-values shown; Missing answers not shown (this occurred <1.5% of all questions)

^2^Other maternal testing answers were test based on symptoms OR a scheduled admission for delivery (n = 4), test for a scheduled admission (n = 4), “based on obstetrical practice” (n = 1), and “universal test at 38 weeks” (n = 1)

^3^Other location of newborn care answers were if mother asymptomatic and desires same room and if mother symptomatic separate rooms (n = 3)

^4^Other newborn testing answers were “test infant if symptomatic” (n = 1), decision made on case by case basis by provider (n = 1) or infection control (n = 1)

**Additional File B. Breastfeeding-Related Practices Among Covid-19 Positive Mothers with Healthy, Term Infants According to Baby-Friendly Hospital Status^1^**

|  | **Baby-Friendly** | **Not Baby-Friendly** | **p-value** |
| --- | --- | --- | --- |
| Overall, n (%) | 114 (35.8%) | 204 (64.2%) |  |
| **Location of newborn care** |  |  | 0.230 |
| Separate room from mother | 34 (29.8%) | 63 (30.9%) |  |
| Same room as mother with precautions to maintain separation | 15 (13.2%) | 40 (19.6%) |  |
| Same room as mother with no precautions | 0 (0.0%) | 1 (0.5%) |  |
| Decisions based on shared decision making on a case-by-case basis | 64 (56.1%) | 97 (47.5%) |  |
| Other^2^ | 0 (0.0%) | 3 (1.5%) |  |
| **Skin-to-skin care in first hour after birth** |  |  | 0.745 |
| Prohibited | 14 (12.3%) | 26 (12.7%) |  |
| Discouraged | 37 (32.5%) | 77 (37.7%) |  |
| Encouraged with precautions | 14 (12.3%) | 19 (9.3%) |  |
| Encouraged with no precautions | 0 (0.0%) | 2 (1.0%) |  |
| Decisions based on shared decision making on a case-by-case basis | 48 (42.1%) | 79 (38.7%) |  |
|  |  |  |  |
| **Delayed or timed cord clamping** |  |  | 0.352 |
| Yes | 78 (68.4%) | 129 (63.2%) |  |
| No | 36 (31.6%) | 75 (36.8%) |  |
| **Early baths (<4 hours)** |  |  | 0.331 |
| Yes | 90 (78.9%) | 170 (83.3%) |  |
| No | 24 (21.1%) | 34 (16.7%) |  |
| **Approach to direct breastfeeding** |  |  | 0.543 |
| Prohibited | 5 (4.4%) | 10 (4.9%) |  |
| Discouraged, but permitted if family strongly desires | 33 (28.9%) | 68 (33.3%) |  |
| Encouraged with precautions | 15 (13.2%) | 34 (16.7%) |  |
| Decisions based on shared decision making on a case-by-case basis | 61 (53.5%) | 92 (45.1%) |  |
| **Approach to expressed breast milk (check all that apply)** |  |  |  |
| May be given by the mother with precautions | 67 (58.8%) | 122 (59.8%) | 0.857 |
| May be given by another caregiver | 92 (80.7%) | 153 (75.0%) | 0.246 |
| Discouraged | 0 (0.0%) | 3 (1.5%) | 0.193 |

^1^Chi-square p-values shown; Missing answers not shown (this occurred <1.5% of all questions)

^2^Other location of newborn care answers were if mother asymptomatic and desires same room and if mother symptomatic separate rooms (n = 3)
