# Supplementary material for: Variation in United States COVID-19 newborn care practices: results of an online physician survey
Source: BMC Pediatr. 2022 Jan 21;22:55. doi: 10.1186/s12887-022-03129-0 (PMC8776982; doi:10.1186/s12887-022-03129-0)
Supplement: Supplementary file 2 — Additional file 2. [file 12887_2022_3129_MOESM2_ESM.pdf]

U.S. National COVID-19 Newborn Practice Survey

You are being asked to voluntarily participate in a research study. The goal of the study is to understand regional differences in obstetric and newborn care practices during the COVID-19 pandemic. The survey can be accessed by the link below. The survey takes approximately 5 to 7 minutes and we ask short questions about obstetric and newborn care practices. We will not record your name or any information that shows your identity. You will not be signing the survey.

We plan to study practices at the hospital-level. This means that we will group responses by all respondents that work at a given hospital. If there are discrepant answers, we will reach out to a clinical leader at your hospital to clarify.

At the end of the survey, we have a link to enter your email address only so that we may give you the results of the survey if you desire. Your email address will not be linked to your survey responses and will be deleted after we send the results.

If you have any questions, please contact Dr. Margaret (Meg) Parker, a neonatologist at Boston Medical Center; margaret.parker@bmc.org; 617-955-0434

Please respond regarding the obstetrical and newborn practices at your hospital during the COVID-19 pandemic. We recognize that practices are changing rapidly at many hospitals. Please respond according the practices performed from Sunday, May 17th- Sunday, May 24. If you work at more than one hospital, please respond regarding the one hospital that you work at the most.

Questions about you:

1. Do you work in a birth hospital?
   - ◯ Yes
   - ◯ No

Thank you, we are interested in birth facilities in this practice survey. End survey.

2. Do you provide direct patient care or oversee direct patient care of newborns?
   - ◯ Yes
   - ◯ No

Thank you, we are interested in the experiences of newborn providers in this survey. End survey

3. What type of provider are you? (check all that apply)
   - ☐ Nurse
   - ☐ Advanced practice provider (PA, NP, APP)
   - ☐ Neonatologist
   - ☐ Newborn Hospitalist/ General Pediatrician
   - ☐ Trainee (fellow, resident)
   - ☐ Other

3a. What other provider type are you? ______________________________

Questions about your hospital:

4. What state is your hospital located in?

◯ Alabama
◯ Alaska
◯ Arizona
◯ Arkansas
◯ California
◯ Colorado
◯ Connecticut
◯ Delaware
◯ Florida
◯ Georgia
◯ Hawaii
◯ Idaho
◯ Illinois
◯ Indiana
◯ Iowa
◯ Kansas
◯ Kentucky
◯ Louisiana
◯ Maine
◯ Maryland
◯ Massachusetts
◯ Michigan
◯ Minnesota
◯ Mississippi
◯ Missouri
◯ Montana
◯ Nebraska
◯ Nevada
◯ New Hampshire
◯ New Jersey
◯ New Mexico
◯ New York
◯ North Carolina
◯ North Dakota
◯ Ohio
◯ Oklahoma
◯ Oregon
◯ Pennsylvania
◯ Rhode Island
◯ South Carolina
◯ South Dakota
◯ Tennessee
◯ Texas
◯ Utah
◯ Vermont
◯ Virginia
◯ Washington
◯ Washington DC
◯ West Virginia
◯ Wisconsin
◯ Wyoming
◯ Puerto Rico

5. What city is your hospital located in?

____

6. What is the full name of your hospital?

____

7. To your knowledge, what is the highest level of neonatal care provided at your hospital, according to the categories designated by the AAP/ACOG Guidelines for Perinatal Care, 2017?

- ◯ Level 1 (basic care for healthy newborns ≥35 weeks' gestation)
- ◯ Level 2 (Level 1 capabilities and specialty care for infants ≥32 weeks or ≥1500 grams with physiologic immaturity or who are moderately ill; mechanical ventilation ≤24 hours or CPAP)
- ◯ Level 3 (Level 2 capabilities and subspecialty care including sustained life support for infants < 32 weeks and < 1500 grams, and all critically ill infants and prompt access to a range of pediatric subspecialists)
- ◯ Level 4 (Level 3 capabilities and location within an institution able to provide surgical repair of complex congenital or acquired conditions and with immediate on-site access to pediatric subspecialists

Please respond regarding practices at your hospital this week.

Obstetric and Delivery Practices:

8. What is the approach to COVID-19 testing of pregnant women prior to anticipated delivery at your hospital? This includes women presenting to L&D in labor as well as women planned for admission for c-section or induction of labor.

- ◯ Universal testing to all pregnant women regardless of signs and symptoms
- ◯ Testing for pregnant women based on signs and symptoms
- ◯ Testing is not routinely available for pregnant women at our facility
- ◯ Other

8a. If other, what is your testing approach?

____

9. What kind of personal protective equipment (PPE) is recommended to be worn by pediatric providers attending vaginal deliveries of COVID-19 positive pregnant women? (Check all that apply)

- ☐ N95 mask
- ☐ Regular surgical mask
- ☐ Eye protection
- ☐ Cap
- ☐ Gown
- ☐ Gloves
- ☐ Other

9a. If other, what kind of PPE is recommended to be worn?

____

10. What kind of personal protective equipment (PPE) is recommended to be worn by pediatric providers attending vaginal deliveries of pregnant women that are NOT COVID-19 positive and DO NOT have signs or symptoms of COVID-19? (Check all that apply)

- ☐ N95 mask
- ☐ Regular surgical mask
- ☐ Eye protection
- ☐ Cap
- ☐ Gown
- ☐ Gloves
- ☐ Other

10a. If other, what PPE is recommended to be worn?

____

11. What is the approach to support persons accompanying pregnant women on labor and delivery (L&D) at your hospital?

- ◯ No support persons may be present on L&D
- ◯ Only 1 support person may be present on L&D
- ◯ 2 or more support persons may be present on L&D

12. What is the approach to testing asymptomatic support persons of COVID-19 positive mothers?

- ◯ We don't offer or require any testing of asymptomatic support persons at this time
- ◯ We offer some testing to support persons
- ◯ We require all support persons to be tested

Newborn Care:

13. What is the preferred approach for location of newborn care for a healthy, term newborn born to a COVID-19 positive mother at your hospital?

- ◯ Care of the mother and infant in separate rooms
- ◯ Care of the mother and infant in the same room with some precautions to maintain separation (e.g. crib 6 feet away, curtain or barrier between mother and infant)
- ◯ Care of the mother and infant in the same room, with no precautions
- ◯ Decisions about location of mother and infant care are based on shared decision making with the mother on a case-by-case basis
- ◯ Other

13a. Other preferred location:

____

14. What is your personal preferred approach for location of newborn care for a healthy, term newborn born to a COVID-19 positive mother at your hospital (regardless of the practice at your hospital)?

- ◯ Care of the mother and infant in separate rooms
- ◯ Care of the mother and infant in the same room with some precautions to maintain separation (e.g. crib 6 feet away, curtain or barrier between mother and infant)
- ◯ Care of the mother and infant in the same room, with no precautions
- ◯ Decisions about location of mother and infant care are based on shared decision making with the mother on a case-by-case basis
- ◯ I don't have a particular personal opinion on this
- ◯ Other

14a. Other personal preferred approach to location of newborn care:

____

15. What is the approach to skin-to-skin care in the first hour after birth for a healthy, term infant born to a COVID-19 positive mother at your hospital?

- ◯ Prohibited
- ◯ Discouraged
- ◯ Encouraged with precautions
- ◯ Encouraged with no precautions
- ◯ Decisions about skin-to-skin care are based on shared decision making with the mother on a case-by-case basis

16. Does your hospital generally perform delayed or timed cord clamping for a healthy, term infant born to a mother that is NOT COVID-19 positive and DOES NOT have signs or symptoms of COVID-19?

- ◯ Yes
- ◯ No

17. Does your hospital generally perform delayed or timed cord clamping for a healthy, term infant born to a COVID-19 positive mother?

- ◯ Yes
- ◯ No

18. Is your hospital performing early baths (< 4 hours) for healthy, term infants born to mothers that are NOT COVID-19 positive and DO NOT have signs or symptoms of COVID-19?

- ◯ Yes
- ◯ No

19. Is your hospital performing early baths (< 4 hours) for healthy, term infants born to COVID-19 positive mothers?

- ◯ Yes
- ◯ No

20. What is the approach to direct breastfeeding among healthy, term infants born to COVID-19 positive mothers at your hospital?

- ◯ Direct breastfeeding is prohibited
- ◯ Direct breastfeeding is discouraged, but permitted if family strongly desires
- ◯ Direct breastfeeding is encouraged with precautions
- ◯ Decisions about direct breastfeeding are based on shared decision making with the mother on a case-by-case basis

21. What is the approach to provision of expressed mother's breast milk among healthy, term infants born to COVID-19 positive mothers at your hospital? (I.e. milk given in a bottle or syringe but not direct breastfeeding) (Check all that apply)

- ☐ Infants may be fed expressed mother's breast milk by the mother with precautions
- ☐ Infants may be fed expressed mother's breast milk by another caregiver
- ☐ Feeding infants expressed mother's breast milk is discouraged

22. Consider the following scenario: A healthy, term infant was born by cesarean section to a COVID-19 positive mother and is anticipated to be discharged on day 3 or 4. What would be the approach to PCR testing of the infant at your hospital?

- ◯ Although in-patient testing is available at our hospital, we generally do not test newborns
- ◯ Testing is not available for infants
- ◯ We do 1 test
- ◯ We do 2 tests
- ◯ More than 2 tests
- ◯ Unsure
- ◯ Other

22a. If other, what is your approach?

____

22b. How many hours after birth is the first infant PCR test usually performed?

- ◯ Before 24 hours
- ◯ Around 24 hours
- ◯ Between 24-48 hours
- ◯ Around 48 hours
- ◯ After 48 hours

22c. How many hours after birth is the second infant PCR test usually performed?

- ◯ Between 24-48 hours
- ◯ Around 48 hours
- ◯ After 48 hours

How have the following newborn hospitalization discharge processes changed among healthy, term infants born to COVID-19 positive mothers at your hospital?

		Have not changed our process	Changed process, but occurs during newborn hospitalization	Changed process and deferred until after discharge
23.	Hepatitis B vaccination	◯	◯	◯
24.	Bilirubin checks	◯	◯	◯
25.	Newborn screening	◯	◯	◯
26.	Critical Congenital Heart Disease screening	◯	◯	◯
27.	Circumcisions	◯	◯	◯
28.	Hearing screening	◯	◯	◯
29.	Red reflex	◯	◯	◯

30. In general, has the timing of discharge of NON-COVID-19 positive mother-infant dyads changed at your hospital?
   - ◯ No, timing of discharge hasn't really changed
   - ◯ Some mother-infant dyads are being discharged early
   - ◯ Many mother-infant dyads are being discharged early
   - ◯ All mother-infant dyads are being discharged early unless there is a medical contraindication

31. What is the approach to visitation of NON-COVID-19 positive mother-infant dyads during the newborn hospitalization at your hospital?
   - ◯ No support persons are allowed to visit mothers and infants in the postpartum area
   - ◯ 1 support person may visit mothers and infants in the postpartum area
   - ◯ 2 or more support persons may visit mothers and infants in the postpartum area

32. Do you have any comments about obstetric and/or newborn care practices at your hospital during the COVID-19 Pandemic?

   ____
